# Application of Hydroxyapatite Obtained by Different Techniques: Metabolism and Microarchitecture Characteristics (Review)

**DOI:** 10.17691/stm2024.16.6.06

**Published:** 2024-12-27

**Authors:** V.A. Markelov, K.V. Danilko, V.A. Solntsev, S.V. Pyatnitskaya, A.R. Bilyalov

**Affiliations:** PhD Student, Institute of Biochemistry and Genetics; Ufa Federal Research Center, Russian Academy of Sciences, 71 Oktyabr Avenue, Republic of Bashkortostan, Ufa, 450054, Russia; Junior Researcher, Laboratory of Cell Cultures, Institute of Fundamental Medicine; Bashkir State Medical University, 3 Lenin St., Republic of Bashkortostan, Ufa, 450008, Russia; PhD, Associate Professor, Department of Biology, Acting Head of Cell Culture Laboratory, Institute of Fundamental Medicine; Bashkir State Medical University, 3 Lenin St., Republic of Bashkortostan, Ufa, 450008, Russia; Junior Researcher, Cell Culture Laboratory, Institute of Fundamental Medicine; Bashkir State Medical University, 3 Lenin St., Republic of Bashkortostan, Ufa, 450008, Russia; PhD, Associate Professor, Department of Internal Diseases; Senior Researcher, Cell Culture Laboratory, Institute of Fundamental Medicine; Bashkir State Medical University, 3 Lenin St., Republic of Bashkortostan, Ufa, 450008, Russia; PhD, Associate Professor, Department of Traumatology and Orthopedics with a Course of the Institute of Continuing Professional Education; Bashkir State Medical University, 3 Lenin St., Republic of Bashkortostan, Ufa, 450008, Russia

**Keywords:** hydroxyapatite, bone grafting, hydroxyapatite metabolism, microarchitecture

## Abstract

The literature reports on microarchitecture and metabolism characteristics of synthetic hydroxyapatite obtained by different techniques were analyzed. The direct relation between hydroxyapatite production process and its microarchitecture was stated to exist. In turn, hydroxyapatite microarchitecture largely specifies its metabolism characteristics (a number of processes related to calcium and phosphorus metabolism). Therefore, with reference to the metabolism of synthetic hydroxyapatite with various microarchitectures, we analyzed the relationship of the material under study with the immune system cells.

Particular emphasis was given to the relationship of hydroxyapatite characteristics with a recipient’s immune system due to the material microarchitecture. The review assessed the possible participation of cell mitochondria in synthetic hydroxyapatite metabolism. There were compared the findings of a recipient’s immune system *in vivo* and *in vitro* depending on hydroxyapatite nanoscale morphology.

The review conclusions emphasized the necessity for further investigations of immunologically mediated metabolism of hydroxyapatite intended for bone implants, including the development of research methods *in vitro* for deeper understanding of the material properties. There was demonstrated the synthetic hydroxyapatite potential in treating bone defects and specified the significance of *in vivo* studies to develop bone surgery and reconstructive medicine.

## Introduction

Current medical technologies dealing with bone tissue regeneration improvement are based on the extensive application of both hydroxyapatite-based grafts and implants. The variety of the materials used reflects a great number of pathological conditions of bone tissue. According to the source, grafts are divided into autologous (autogenic), allogenic, and xenogeneic. In turn, the implants for bone regeneration can be conditionally divided by the material origin used for their production. The key value for bone implant production is synthetic hydroxyapatite made using chemical synthesis. However, there are many materials based on processed hydroxyapatite of biogenic origin obtained using various processing techniques.

The use of autologous bone grafts is a recognized gold standard of bone grafting. In clinical practice autologous transplantation has already been usefully employed for a century [1, 2]. However, despite the fact that autologous material is standard, the number of studies aimed at searching for some alternative materials keeps growing. It is due to critical drawbacks of the methods used to obtain autologous material: firstly, limited volume of transplantation material [3, 4]; secondly, possible complications on a donor’s site [5, 6]. A general list of complications includes infections, hematomas, chronic pain, fractures, as well as vessel and nerve damage [7]. The volume of the material taken out correlates with the complication risk [8] significantly limiting the use of autologous bone grafting. Therefore, it is extremely important to use alternative transplant materials, among them there are allogenic [9–11] and xenogeneic [10, 12]; that is why they under extensive study.

Allogenic material is functionally the most approximate one to autologous transplant material among those mentioned above. Its main advantage is relatively high availability [13]. Therefore, it has been widely used for long years in clinical practice to reconstruct extensive bone injuries. Like autogenous, allogeneic transplant material has a high degree of similarity to the native bone structure. It has similar mechanical properties, as well as osteoinductive and osteoconductive properties, and to some extent it is biocompatible. The specified properties are limited due to the necessity for the transplant material decellularization [11]. According to some authors, the main problem of the method is the absence of integrated protocols and a potential risk of transmitting infectious diseases [14].

The category of xenogeneic bone grafts has a number of similarities to allogenic materials. For instance, there are evidences confirming high osteoconductive properties of xenogeneic bovine bone material [15]. However, the independent use of xenogeneic graft, despite an uneventful postoperative period in certain cases [16], shows low quality of clinical results [17, 18]. The main negative results of xenotransplantation are fibrous graft encapsulation [19], vicious union, and pain syndrome [20]. Moreover, due to extremely high duration of xenograft integration (57 weeks) compared to an allograft (16 weeks), many experts cast doubt on the possibility of independent usage of xenogeneic grafts [17].

Thus, it is still urgent to solve the problem of standardization and high risk of transmitting infectious agents when transplanting allogenic [21, 22] or xenogeneic [23] bone materials. High risk of an increased immune response in allogenic and xenogeneic bone grafting is needed to be taken into consideration [24, 25]. The presented problems in using allogenic and xenogeneic grafts cause the necessity for developing safer, more available, and comparatively efficient alternative techniques.

Therefore, it is reasonable to use synthetic hydroxyapatite as a base for grafts, which enable them to take on the role of functional alternatives to bone grafts. It is proved by the experience of clinical use of hydroxyapatite [26–29]. Its popularity can be explained by the fact that hydroxyapatite is a native form of bone tissue calcium, it occupying 70–90% of its matrix volume. In bone tissue, hydroxyapatite is in the form of small-sized crystals and characterized by a stoichiometric formula: Ca10(PO4)6(OH)2 [30]. Special attention is drawn by a composite form of using synthetic hydroxyapatite, since the native bone is also a composite structure [31, 32].

Hydroxyapatite was shown to contribute to bone tissue regeneration providing favorable osteoimmune microenvironment [33]. However, even if the materials most suitable for obtaining bone grafts are used, a preliminary detailed analysis of an immune response is required. Within the given context, a number of unique immunological parameters of hydroxyapatitebased materials can acquire great importance [34, 35]. Modern literature data [33, 36, 37] demonstrate an immunomodulating effect of hydroxyapatite-based materials. So, there was studied a macrophagemediated regenerative effect of hydroxyapatite related to the graft material metabolism [33]. Such information enables to manipulate these parameters adjusting structural and textural material characteristics, as well as including various functional components. The relationship of synthetic hydroxyapatite nanostructural parameters and its immunomodulating properties is still an open issue [33]. There are left unclarified the hydroxyapatite metabolism parameters on a cell level; and the role of monocytes/macrophages in particular [33, 38].

## Sourcing methodology

The literature for the present review was searched in MEDLINE (PubMed) and Google Scholar by key words and their combinations: hydroxyapatite bone grafts, hydroxyapatite nanoparticles, nanostructured hydroxyapatite, bone grafts for biomedical applications, hydroxyapatite synthesis for bone grafts, hydroxyapatite for biomedical applications, hydroxyapatite production for bone grafting, biogenic hydroxyapatite, dry method hydroxyapatite production, dry method of hydroxyapatite production, chemical method of hydroxyapatite production, osteoclast response to synthetic hydroxyapatite, response to synthetic hydroxyapatite, properties of nanostructured hydroxyapatite, osteogenic potential of hydroxyapatite, osteoconductive potential of hydroxyapatite.

Available scientific data was gained till November 28, 2023. The articles were selected by two coauthors, independently from one another using manual search. All differences were smoothed by means of discussions by an authoring team, as well as by consulting the third expert. A total of 133 scientific articles were selected.

## Synthetic hydroxyapatite production processes and synthetic hydroxyapatite nanostructure characteristics

There are two main categories of preparation techniques: solid-phase methods and those using solvents [39].

**Figure 1.**
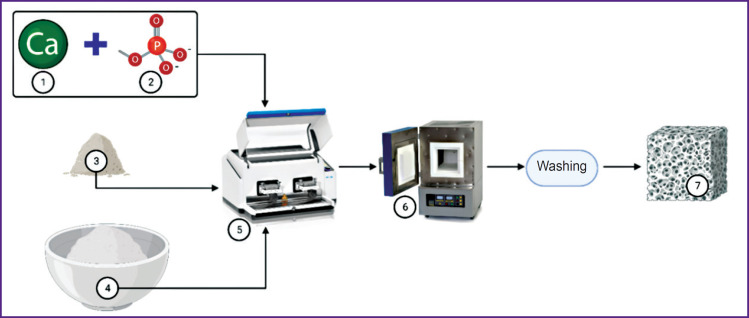
Algorithm of solid-phase method of hydroxyapatite production: *1* — source of calcium raw material (calcium hydroxide); *2* — source of phosphates (ammonium hydrophosphate); *3* — optional introduction of salts; *4* — prepared calcium phosphate powder; *5* — intensive mechanical grinding (vibrating ball mill); *6* — high-temperature treatment of the mixture; *7* — transferring material in the form of primary raw material. The illustration was created using the online tool BioRender (https://www.biorender.com/)

***Solid-phase methods*** are characterized by using a mechanical action and relatively high temperatures (Figure 1).

The methods require no solvents [40, 41]. A solidphase technology has low sensitivity to production conditions [42, 43] and generates a product with high crystallization [39, 44]. However, such hydroxyapatite often includes intermediate phases [45] and exhibits low biomimetic properties [46]. On the other hand, such material production is easily scaled using the optimal temperature of 1050°C. So, high temperatures for hydroxyapatite production slightly decrease its porosity [45], it being the significant restriction of its usage as a material for bone grafting.

**Figure 2.**
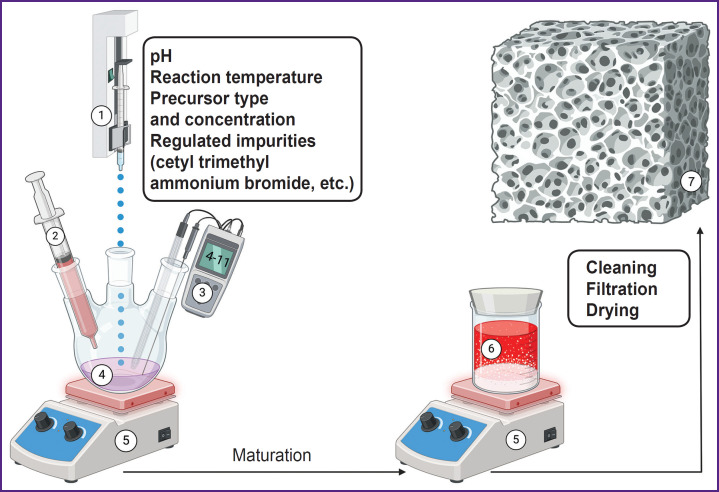
Algorithm for obtaining hydroxyapatite by chemical deposition: *1* — a syringe pump with Ca^2+^ reagent (calcium nitrate Ca(NO_3_)_2_); *2* — pH controller (ammonium solution); *3* — pH meter; *4* — phosphate anions (diammonium phosphate (NH_4_)_2_HPO_4_); *5* — stirring and temperature control; *6* — precipitation of hydroxyapatite particles; *7* — transfer material in the form of primary raw material. The illustration was created using the BioRender online tool (https://www.biorender.com/)

***Chemical deposition methods*** are characterized by using solvents — the sources of calcium and phosphates [39] — in the presence of additives [47–50] in acid or basic media (Figure 2). The range of production conditions of these methods is extremely diverse: there is a great variability of pH values (3–12) [51, 52] and temperatures (25–90°C) [39, 53].

Precipitation enables to obtain hydroxyapatite particles with native morphology (needle-like) [54] and makes it possible to manipulate it [40]. Precipitation provides the preparation of the material with desired ionsubstitution by magnesium [47], strontium, lithium [55], manganese [48], aluminium [49], zinc [55, 56], selenium [50], and other metals [57, 58].

Chemical deposition is used to obtain a composite material [59] forming the coating for polymer [60–62], metal [63, 64], and combined scaffolds [65, 66]. Through this process, there can be obtained composite porous micelles [67], nanoparticles [68], nanotubes [69], and nanorods [70]. Hydroxyapatite obtained through chemical deposition has low crystallinity [40]. Despite chemical deposition requires no high temperatures, the method needs the strict control of synthesis conditions. On the one hand, it decreases hydroxyapatite production scaling by the method, although, on the other hand, it enables the fine adjustment of the hydroxyapatite morphology and nanoparticle size [53]. It is likely to be an important advantage when using the method in research practice.

***An electrochemical method*** is based on aqueous solutions [71]. The technique enables to form a uniform coating at moderate temperatures, providing strong integration of hydroxyapatite into porous agents [71, 72]. A striking example is the method of impulse electrodeposition, which decreases the release of gaseous hydrogen, improving hydroxyapatite integration [71]. Similarly to chemical deposition, electrodeposition is used to produce composite structures of hydroxyapatite with the most diverse morphology and composition [73]. Such structures can include different alloys [74–76], including aluminium ones [77], and polymer bases [78]. The abovementioned electric deposition also has morphological variety: hydroxyapatite nanotubes [79], nanoparticles [80], and other disperse hydroxyapatite forms [81, 82, 57].

**Figure 3.**
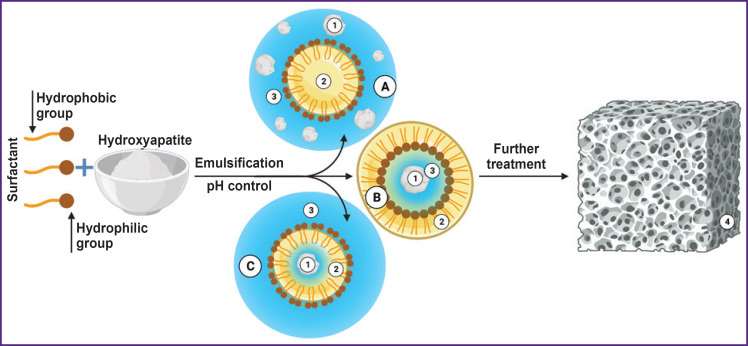
Algorithm for hydroxyapatite production using emulsion method: *1* — hydroxyapatite particles; *2* — oil phase; *3* — aqueous phase; *4* — implantation material; *A* — oil encapsulated in an aqueous phase containing particles; *B* — oil encapsulates an aqueous phase with a hydroxyapatite particle; *C* — emulsion system, where an aqueous phase contains the oil with an encapsulated aqueous medium containing hydroxyapatite particle. The illustration was created using the BioRender online tool (https://www.biorender.com/)

***Emulsion method*** belonging to category 2 methods is one of the most effective for obtaining nanostructural hydroxyapatite powder (Figure 3). Powder particles form in a disperse medium of two immiscible solvents stabilized by surface-active agents (SAA). The way the emulsion is produced is determined by SAA nature and concentration [83]. An emulsion provides a favorable medium to regulate particle growth. In turn, hydrophobic SAA are easily removed by ignition [84, 85].

The emulsion method advantage is the strict control of morphological parameters of nanoparticles, and due to this the technique is often used to obtain porous materials. The sources of calcium and phosphate can be calcium nitrate and phosphoric acid. As SAA, there can be used dioctyl sodium sulfosuccinate, dodecyl phosphate, polyoxyethylene, non-polyphenol ether, polyoxyethylene ether, cetyl trimethyl ammonium bromide, and sodium dodecyl sulfate. In addition to SAA characteristics, end parameters of hydroxyapatite can be determined by temperature, water and organic phase relationship, pH and precursors concentration [39].

**Figure 4.**
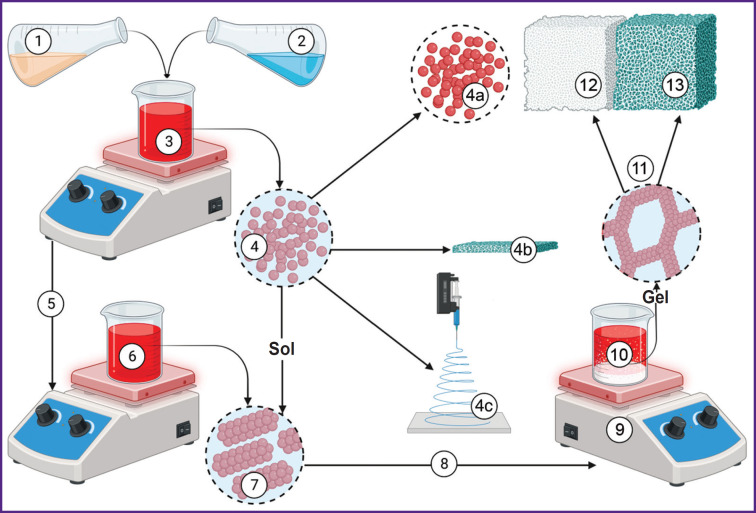
Sol-gel method operation scheme: *1* — phosphate-containing reagent (phosphorus pentoxide); *2* — calcium-containing reagent (calcium nitrate); *3* — solvent (water or ethanol), control of sol formation reaction; *4* — sol condition of the reaction mixture (*4а* — deposition with the material formed as powder; *4b* — formation of coatings; *4c* — formation of ceramic fibers); *5* — the reaction mixture transfer from sol to gel; *6* — control of coagulation reaction parameters; *7* — coagulation; *8* — direct transfer into gel-formation condition; *9* — control over gel-formation reaction conditions; *10* — gel; *11* — evaporation and extraction by a dissolvent; *12* — aerogel formation; *13* — dense ceramics. The illustration was created using the BioRender online tool (https://www.biorender.com/)

***Sol-gel method*** is a relatively popular method to obtain hydroxyapatite (Figure 4). As precursors, there can be used calcium chloride and various organic phosphites [86]. It is convenient for obtaining film coatings [87, 88] and aerogel structures [39]. The technique presupposes precursors hydrolysis with the formation of micelles associated with templates in aqueous or organic media. It provides high chemical homogeneity of hydroxyapatite [89], appropriate stoichiometry, and minimal size clustering. There were additionally indicated high rate of surface-specific area and available mesoporous volume of hydroxyapatite obtained by the method [90]. *In vitro* studies confirm good biodegradation characteristics of the material obtained by this method [40]. However, it has a limited scaling potential due to low availability of precursors. Moreover, insufficient manufacturing control can promote the formation of secondary phases in the form of CaO, Ca2P2O7, Ca3(PO4)2, and CaCO3 [39].

Each of the methods presented has its advantages; therefore, the most logical step towards improving synthetic hydroxyapatite quality can be the combination of the above-described methods of its production. So, an emulsion method product, for example, undergoes hightemperature treatment [84], which is often a final stage of combined technologies, and enhances the material crystallinity [85]. There are two main variants of hightemperature treatment as separate methods. They are pyrolytic spraying and a sputter coating technique. The first one consists of spraying a solution of calcium and phosphorus salts in a high-temperature furnace followed by water evaporation and the formation of hydroxyapatite crystals. The second technique presupposes high-temperature coating of the target by hydroxyapatite. In both cases the morphology and size of the particles can be regulated, since the parameters directly depend on the characteristics of sprayed and evaporated drops [40].

## Biogenic sources of hydroxyapatite

Let us consider the most common techniques used to produce hydroxyapatite from biogenic sources, their popular raw material is biological waste: great cattle bones [91, 92], egg shell [93, 94], sea organisms [95–98], and plants. The latter can be used to extract hydroxyapatite [99] or as a solvent [100, 101]. In addition, they are used as calcium [102] and phosphate [103, 104] sources. Literature reports demonstrate the applicability of nanostructural hydroxyapatite of plant origin [105] and mycogenous hydroxyapatite [106].

The key requirement for this raw material type is the possibility to remove organic residues, it being achieved through heat treatment [93], subcritical water treatment [107, 108], alkaline thermal hydrolysis [108], and fermentation techniques [109].

Natural hydroxyapatite has different substitution degrees of such elements as Na^+^, Zn^2+^, Mg^2+^, K^+^, Si^2+^, Ba^2+^, F^–^, and CO_3_^2–^ [110]. It explains the multiple roles of native hydroxyapatite of bone tissue. There was described high biomimeticity of the obtained hydroxyapatite and the mineral phase of human bone [93]. It was confirmed by the data indicating the similarity of leading morphological and microarchitectural parameters of hydroxyapatite treated at high temperatures [111]. So, the specific surface and morphology of synthesized hydroxyapatite particles are in the range of the values characteristic for native bone tissue [112–115]. It should be noted that biogenic hydroxyapatite can serve as raw material source for many production methods of synthetic hydroxyapatite (see the Table [92, 93, 95, 96, 98, 115–123]).

**Table:** Application of different biogenic raw material types to obtain hydroxyapatite

Source of raw materials	Method of extraction	Description of an end product (hydroxyapatite)	*In vitro* and *in vivo* study findings	References
Animal bone waste	Ignition	Hexagonal nanoparticles, 300–500 nm in size	Good viability and proliferation of cells	[92, 115]
	Treatment using a ball mill	Nanoparticles, under 500 nm in size	Osteogenic differentiation of dental stem cells	[116]
*M. furnieri* waste	NaOH and H_2_O_2_ treatment, t=800°C	Particles with pores ~8 μm in size	Tissue growing in a graft	[95, 117]
*H. molitrix* bone waste	NaOH and acetone treatment	Powder, average crystallite size ~58.3 nm	MG63 cell viability is 91%	[118]
	Ignition, t=900°C	Powder, average crystallite size ~64.3 nm	MG63 cell viability is 86%	
*Tilapia* bone waste	Ignition, t=600–800°C	Porous grains with high Mg^2+^ substitution degree	High biocompatibility degree	[114]
*E. chlorostigma* bone waste	Alkaline hydrolysis and ignition, t=600°C	Nanoparticles, 29.5 and 82.12 nm in size, respectively	High biocompatibility degree of L929 cells. High remineralization potential	[119]
*L. catla* and *N. japonicus* scales	Ignition, t=800°C and treatment using a ball mill	Porous nanoparticles, 30–60 nm in size, and 10-nm crystallites	In combination with polycaprolactone, there was proliferation and perfect adhesion indices	[98]
*L. lentjan* scale	Hydrothermal treatment, t=280°C	Rods 50–100 nm long, 8–12 nm in diameter, and spheroids 15–50 nm in diameter	Biocompatibility and high osteogenic potential of human mesenchymal stem cells	[120]
Plancton	Leaching of solid particles	Porous nanohydroxyapatite	Adhesion, proliferation, and viability	[121]
*A. glabrata* shells	Ignition, t=900°C and deposition	Nanoscale rods 13.3–15.2 nm	Inhibits development of pathogenic bacteria and fungi	[96]
*Sepia* cuttlefish skeleton	Heat treatment NH_4_H_2_PO_4_, t=200°C	Biomimetic microspheres 1-2 μm	MG63 proliferation. High alkaline phosphatase activity and osteocalcin expression	[122]
*A. fulica* shells	Sintering in the presence of (NH_4_)_2_HPO_4_ In succession: t=150°C (night), 80°C (up to complete drying), 750°C (1 h)	Nanoparticles, 87.7–88.9 nm in size	Antibacterial activity	[123]
Egg shell	H_3_PO_4_ heat treatment In succession: t=80°C (night), 150°C (24 h), 80°C (24 h)	Particles 21.0–40.8 nm containing Mg and Sr	High cell adhesion of MG63 cells	[93]

The presence of a great variety of alternative methods for obtaining synthetic hydroxyapatite using the most diverse precursors provides great opportunities to produce bone grafts with various nanostructural parameters. It is due to the described above dependence of hydroxyapatite nanoarchitecture on its production methods. The nanostructure variety is an important condition for choosing the research and therapeutic strategies for bone graft application.

## Metabolism and interaction characteristics with the recipient’s immune system

Most grafts used for bone tissue regeneration are temporary structures, which provide structural support, contribute to bone repair, and direct bone growth. Semisynthetic and synthetic materials are available and can be modified (for instance, there can be confirmed the positive dynamics of new bone formation based on hydroxyapatite scaffold [124].

Among the most common synthetic bone expletive substances, there is a group of calcium-phosphate ceramics, including hydroxyapatite, β-tricalcium phosphate, and α-tricalcium phosphate, calcium sulphate, as well as bioactive glass and polymers [125]. Hydroxyapatite has a number of characteristics compared to other synthetic bone substitutes. Compared to β-tricalcium phosphate, carbonate-substituted hydroxyapatite [126, 127] exhibits increased solubility under the conditions imitating lacunas of Howship (resorption fossae) [128]. The latter structure is the result of osteoclastic activity and plays an important role in bone remodeling [129]. β-tricalcium phosphate resorption is maximum in physiologically normal conditions [128]. Even higher resorption in physiological conditions was found for α-tricalcium phosphate [130]. In addition, it should be noted that hydroxyapatite substituted by magnesium exhibits lower resorption in bone defects compared to hemihydrate of calcium sulphate [131]. In its turn, calcium sulphate demonstrates incomplete osteogenic response compared to β-tricalcium phosphate/apatite [132, 133].

When comparing hydroxyapatite and bioactive glass, there is a striking osteoinductive response of the latter [125]. It is due to an amorphous layer formed on the glass surface providing the conditions for the concentration of structural proteins and growth factors [134]. A comparative analysis of hydroxyapatite, bioactive glass, and composites containing both materials showed the increase in osteoconductive potential when hydroxyapatite was added [135]. Apart from that, the grafts based on composite materials made of hydroxyapatite and bioactive glass exhibit higher mechanical stability after implantation compared to a pure bioglass material [135, 136].

The comparison of hydroxyapatite grafting and bioactive glass grafting demonstrates the greater area of neoformed bone and the greater number of TRAPpositive (TRAP — tartrate-resistant acid phosphatase) cells when using hydroxyapatite [137]. TRAP-positive cells are mostly presented by osteoclasts and macrophages [138, 139].

The distinctive feature of hydroxyapatite behavior under bone remodeling against the background described in the works [137, 139] can be a particular relation of the material with osteoclastic activity.

As for polymer synthetic bone substitutes, there is a similar tendency for osteogenic potential increase under conditions of including hydroxyapatite into their composition [140]. When there is used the polyurethane composite with 40% hydroxyapatite added, the capacity for *in vitro* biomineralization and osteogenic differentiation increases. Similarly, *in vivo* studies indicated the considerable volume of vascularized bone tissue [141]. There is the same tendency when hydroxyapatite is included in polyethylene glycol diacrylate composition: the improved mechanical properties and biocompatibility are exhibited [142, 143].

The data presented suggest the interaction of hydroxyapatite with TRAP-positive cells [137, 144–146], in particular, with osteoclasts and their immune precursors [138, 139]. The relationship between hydroxyapatite and a marked acute immune response in recipients is confirmed by aseptic destruction and bone tissue osteolysis in response to the material implantation. The response directly depends on the presence of hydroxyapatite particles, under 53 μm in size, decreasing the viability of osteoblasts and osteoclasts [147]. The mentioned response to small-sized hydroxyapatite is characteristic for different cells, including tumor ones; hydroxyapatite particles inhibit their proliferation due to protein synthesis inhibition, blocking the accessibility of ribosomes for mRNA [148]. In addition, it should be noted that nanosized hydroxyapatite initiates selective apoptosis [36] and blocks melanoma growth [149]. It is foremost related to the disturbed cell homeostasis of calcium and the activation of endogenic mitochondrial stimuli of apoptosis [81] that seems intriguingly in the scope of hypothesis of mitochondrial mineralization of bone tissue [38]. Moreover, hydroxyapatite enables to initiate monocytes flattening and the differentiation of macrophages into osteoclast-associated phenotype. Hydroxyapatite effect stimulates the expression of a nuclear factor kappa B ligand and the podosome belt formation in monocytes/macrophages, the activity of osteoclasts being modulated [150, 151].

It should be noted that the hydroxyapatite treated using the method of solution and deposition compared to untreated hydroxyapatite promotes TRAP-positive staining area growth. The index is associated with the osteoclastic activity. The differences under observation are explained by the presence of the nanoscale hydroxyapatite in untreated material, whereas untreated material exhibits coarse-grain structure [152]. It is in good agreement with the fact that plate-like nanostructure of hydroxyapatite is associated with active cell proliferation at early co-incubation stages. On the contrary, for hydroxyapatite with needle-like nanostructure, high cell proliferation is found only at late experiment stages. Plate-like hydroxyapatite microstructure is also related to the greater number of flattened macrophages [153].

There was procured valid evidence in favor of the effect hydroxyapatite particle morphology has on cytokine synthesis by mouse dendritic cells. The highest IL-1β (interleukin 1β) secretion was found in the response to needle-like hydroxyapatite. In contrast, there was found no capability to enhance IL- 1β synthesis for spherical particles, ~100 μm in size. It was expected that in intraperitoneal administration, needle-like hydroxyapatite particles cause the stronger inflammatory response compared to their spherical analogs. In mouse peritoneal exudate cells stimulated by needle-like particles, ~5 μm in size, higher TNF-α (tumor necrosis factor alpha) levels were found in response to re-stimulation. All exudate samples, other than those stimulated by spherical particles, ~100 μm in size, showed decreased IL-10 production. In combination with the dynamics of infiltration by mast cells and macrophages, it indicates the less inflammatory response to large spherical particles compared to needle-like ones [37]. Moreover, the material particle morphology plays a key role in forming osteoconductive properties owing to the material resorption rate regulated by TRAP-positive osteoclast-like cells [154].

Comparatively, later studies also have confirmed the particular importance of the nanoscale morphology of the hydroxyapatite-based graft material. For instance, hydroxyapatite with grooved structure compared to the control hydroxyapatite promotes better macrophage attachment and decreases the production of antiinflammatory cytokines of TNF-α, IL-1β, and IL-6. The phenomenon is due to the decreased accumulation of reactive oxygen species (ROS) owing to the modulation of mitochondrial functions. However, no effect on the character and dynamics of macrophage polarization was revealed [155], although the later studies have reported on such a possibility [35]. On the other hand, in case of nanostructural hydroxyapatite action on macrophages, there is an increase in the synthesis of TNF-α, IL-6, adenosine triphosphate, nicotinamide adenine dinucleotide, and ROS [156]. At the same time, CD8-positive T-cells demonstrate increased expression of IFN-γ and CD107α [157]. In contrast, micro-grooved structure decreases IL-6 expression owing to inhibiting miR-214, and thus contributing to the survival of mesenchymal bone marrow stem cells [155]. The rod-like hydroxyapatite ability to have an effect on mitochondrial functionality is proved by its antitumor action mechanism. For example, in nano-rod hydroxyapatite internalization, mitochondrial ROS and cathepsin B are released [157]. The hydroxyapatite with the mentioned morphology demonstrates marked immunomodulating [33, 36] and proapoptotic [36] properties.

Next literature example [148] demonstrates the difference in the properties of hydroxyapatite with different morphology under *in vivo* and *in vitro* conditions. *In vivo* studies showed a comparable osteogenic potential for both nanostructural and submicron hydroxyapatite. Moreover, nanostructural hydroxyapatite exhibits greater osteogenic potential. Concurrently, the authors emphasized the relationship of osteogenesis and osteoclastogenesis. However, in *in vitro* experiments nanostructural hydroxyapatite has an inhibiting effect concerning the early differentiation and survival of osteoclasts. It decreases the expression of specific markers of osteoclastogenesis, as well as TRAP activity, including ROS-generating activity. Therefore, it is noteworthy that there are reports on ribosomal and mitochondrial mechanisms of inhibiting cell activity by hydroxyapatite [36, 148]. Meanwhile, submicron hydroxyapatite in *in vitro* experiments is able to have a stimulating effect in relation to osteoclast differentiation and activity [133].

To conclude, it is important to note Ca^2+^ content analysis indicates decreased osteoclastogenesis at early incubation stages of RAW 264.7 cells with nanostructural hydroxyapatite. However, on day 14 the researchers observed the gradual increase and sustaining this osteoclastogenesis characteristic. However, in a similar experiment for submicron hydroxyapatite, on day 14 there was the registered steep downfall of this osteoclastogenesis characteristic that can be due to osteoclastic apoptosis.

There are some studies, which have shown the activating properties of nanostructural hydroxyapatite regarding osteoclasts [152]. The observed contradiction can be due to the differences in infiltration parameters of immune cells in *in vivo* and *in vitro* experiments (e.g., the infiltration dynamics of macrophages and mast cells in nanostructural hydroxyapatite grafting [37]). It is not unlikely that the observed phenomenon has ROS-dependent mitochondrial origin and is associated with apoptosis. The implication is that the nature of the phenomenon under study is complex since the effect of differences in the infiltration stability parameters of immune cells is not exclusive of ROSdependent mitochondrial mechanism of osteoclastic apoptosis. Thus, the further advance in the phenomenon comprehension requires integrated studies including the assessment of such molecular mechanisms as ROSdependent mitochondrial apoptotic cascades.

## Conclusion

According to the data presented, synthetic hydroxyapatite as a bone graft material exhibits high osteogenic potential and is capable of stimulating osteoclastic activity. The comparative analysis showed the use of hydroxyapatite as a component of composite materials to enhance mechanical stability and osteoconductive properties of grafts.

There is the hypothesis describing the bone tissue mineralization as the energy-dependent movement of calcium cations and phosphate anions of blood serum into osteoblastic mitochondria followed by the deposition of amorphous microbatches of calcium phosphate. The hypothesis is successfully consistent with the literature data confirming a significant role of mitochondria in the metabolism of both synthetic and native hydroxyapatite. On the other hand, synthetic hydroxyapatite and native hydroxyapatite have a positive effect on mitochondrial ROS-dependent functions. However, the character of such an effect directly depends on hydroxyapatite microarchitecture. The represented facts enable to distinguish the main direction of future research. For instance, it is necessary to reveal certain metabolic mechanisms of synthetic hydroxyapatite of bone grafts by determining the role of mitochondrial apparatus of cells.

The represented literature data make rather complete picture of differences between *in vivo* and *in vitro* study findings of synthetic hydroxyapatite with different nanoscale morphology. Primarily, they enable to conclude that the adequate assessment of hydroxyapatite as an implantation material with nanoscale morphology, as for now, is possible only if there is relatively constant and long-time infiltration of immune cells. These conditions to the full extent can be achieved in *in vivo* studies. However, we are aware of the need for checking the declaration by further target research.

In addition, among the key characteristics of hydroxyapatite as a material for bone grafts, there can be specified its specific character of interacting with monocytes/monophages, osteoclasts, and Т-cells of the recipient’s body. Moreover, this characteristic can be directly regulated by the nanoscale morphology of the material providing the preservation of its macroscopic structure. In this context, particular interest can be provoked by the ability of nanostructural hydroxyapatite to have an effect on ribosomes and mitochondria of many cells including tumor cells. Combined with satisfactory mechanical properties, high scaling potential, and the production process unification, the material can be used to treat major bone defects. It is worth noting separately the defects resulting from tumor removal, that are due to an antitumor effect of nanostructural hydroxyapatite. However, the issue also has to be elaborated using target studies.
